# Perilymphatic fistula with characteristic findings of the inner ear by contrast-enhanced magnetic resonance imaging: a case report

**DOI:** 10.3389/fneur.2023.1276991

**Published:** 2023-10-19

**Authors:** Yusuke Ito, Toru Seo, Yoshiyuki Sasano, Fumihiro Mochizuki, Izumi Koizuka

**Affiliations:** ^1^Department of Otolaryngology, St. Marianna University School of Medicine, Kawasaki, Japan; ^2^Department of Otolaryngology, St. Marianna University Yokohama Seibu Hospital, Yokohama, Japan; ^3^Department of Otolaryngology, University of Miami Miller School of Medicine, Miami, FL, United States

**Keywords:** perilymphatic fistula, magnetic resonance imaging, HYDROPS, vHIT, Reissner's membrane, vestibular atelectasis

## Abstract

A perilymphatic fistula (PLF) presents with abnormal traffic in the otic capsule, causing cochlear and vestibular symptoms. However, the mechanisms underlying symptom recurrence remain controversial. Herein, we report the case of a 27-year-old female who complained of hearing disturbance in her right ear and recurrent vertigo after sudden onset of hearing loss with vertigo. The caloric test revealed unilateral weakness in the right ear, and the video head impulse test (vHIT) showed decreased vestibulo-ocular reflex (VOR) gain. Contrast-enhanced magnetic resonance imaging (MRI) using hybrid of reversed image of positive endolymph signal and negative image of perilymph signal (HYDROPS) indicated a collapsed endolymphatic space. As the vestibular symptoms did not improve, an exploratory tympanotomy was performed on the right ear. Although perilymph leakage was not noted in the oval or round windows, both windows were sealed with connective tissue. The patient's vestibular symptoms rapidly improved after surgery, and postoperative contrast-enhanced MRI showed improvement in the collapsed endolymphatic space. Although the caloric test revealed unilateral weakness, the VOR gain on the vHIT improved to normal on the right side. Thus, these findings indicated that recurrent symptoms caused by PLF are associated with a collapsed endolymphatic space. We speculate that the collapsed endolymphatic space was due to a ruptured Reissner's membrane. We hypothesized that sealing the fistula would promote normalization of perilymph pressure. The ruptured Reissner's membrane may have been gradually repaired as vestibular symptoms improved. This case adds to the existing literature on the occurrence of the “double-membrane break syndrome”. Collapse of the endolymph due to a ruptured Reissner's membrane may be the cause of PLF symptoms.

## 1. Introduction

A perilymphatic fistula (PLF) presents with abnormal traffic in the otic capsule, causing cochlear and vestibular symptoms. PLFs can be classified as those with and without identifiable causes. PLFs are more likely to be diagnosed after middle or inner ear surgery, such as stapes surgery, or trauma. However, it is often difficult to identify the cause of PLFs in the absence of prior surgery or trauma. In Japan, PLFs are classified into four categories according to the cause and diagnosed based on clinical symptoms, microscopic or endoscopic examination of the fistula between the middle and inner ears, or detection of a perilymph-specific protein in the middle ear (Table 1) (1, 2). Sarna et al. proposed similar diagnostic criteria (3). However, the mechanisms underlying symptom recurrence remain controversial. One of the most common signs is pneumolabyrinth, which aids in the diagnosis of Category 1 cases. But, this is not a common finding in cases of Categories 2, 3, and 4 (4). We report a case of PLF that caused hearing loss and vertigo in a young female patient. In this case, we gained insight into the pathogenesis of PLF by performing preoperative and postoperative vestibular function tests and contrast-enhanced magnetic resonance imaging (MRI) using hybrid of reversed image of positive endolymph signal and negative image of perilymph signal (HYDROPS) to identify endolymphatic hydrops in Meniere's disease (MD).

**Table 1:** PLF categorization.

Category 1: linked to trauma, middle and inner ear diseases, middle and/or inner ear surgeries
Category 2: linked to barotrauma caused by antecedent events of external origin (such as flying or diving)
Category 3: linked to barotrauma caused by antecedent events of internal origin (such as straining, sneezing or coughing)
Category 4: has no apparent antecedent event

## 2. Case report

**Figure 1:**
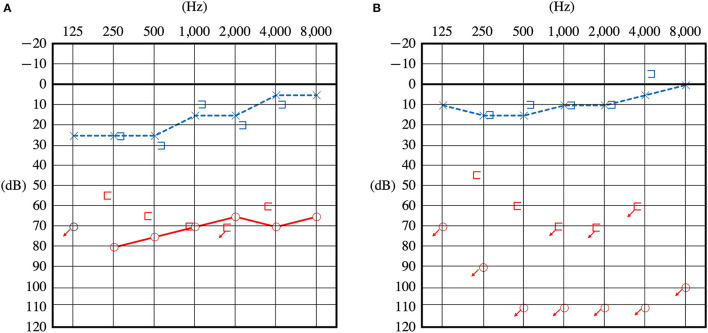
Pure tone audiometry before surgery. Audiometry of the patient upon presentation of sudden hearing loss **(A)** and 1 year later showing a reduction in hearing **(B)**.

A 27-year-old female complained of hearing disturbance in her right ear and recurrent vertigo after sudden onset of hearing loss with vertigo. She had no history of trauma or headaches. At the age of 15 years, she began to experience vertigo preceded by right ear blockage without any trigger. Vertigo was induced by blowing the nose. At the age of 19 years, her symptoms worsened further, and the frequency of vertigo increased; therefore, she visited a local Ear, Nose and Throat (ENT) clinic, but no hearing loss was noted and no apparent abnormality was found. At the age of 26 years, she suddenly developed hearing loss in her right ear and severe vertigo, which she had never experienced before, and visited another ENT hospital. The mean pure-tone air conduction threshold at 500 Hz, 1,000 Hz, and 2,000 Hz was 70.0 dB. An A-B gap was present at low frequencies (Figure 1A). The spontaneous nystagmus turned leftward, especially in the right lateral recumbent position. She was diagnosed with sudden onset hearing loss with vertigo and was treated with prednisolone (60 mg/day tapered for 1 week). However, the patient's hearing did not improve. Variations in low frequencies of 5–10 dB were observed during the monthly audiometric testing. Thereafter, 2–3 times in a month, she experienced recurrent vertigo lasting several hours, with hearing loss, tinnitus, and a blocked sensation in the right ear. During the interictal period, she had gait instability, especially deviating to the right, and dizziness in the right lateral recumbent position.

**Figure 2:**
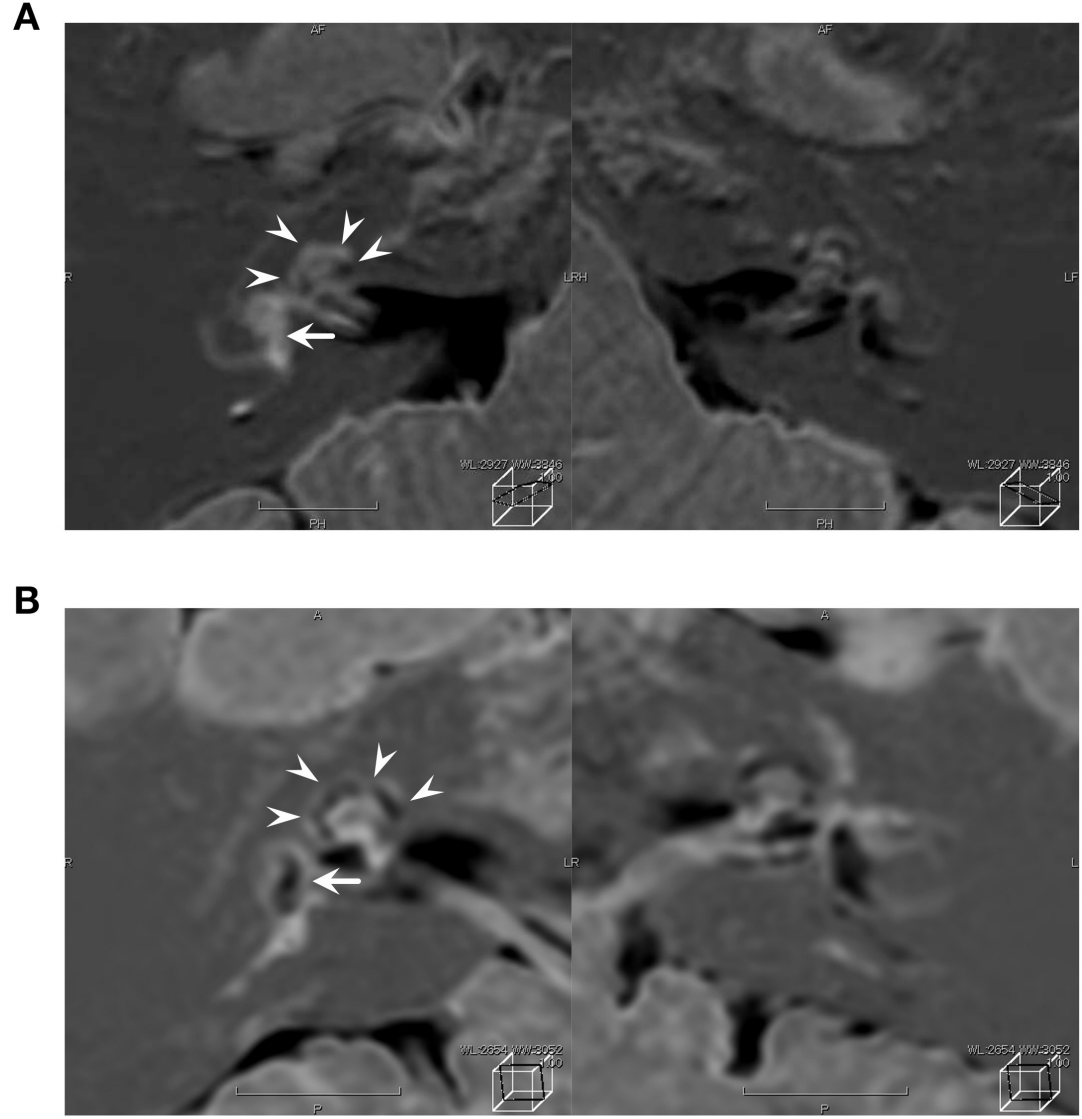
Contrast-enhanced MRI images using HYDROPS. The arrow heads and long arrows indicate the enlarged cochlea vestibule, respectively. Contrast-deficient images (black areas) indicate the presence of endolymph. A contrast-enhanced deficient image of the endolymph of the right ear was rarely observed preoperatively **(A)** but is observed postoperatively **(B)**.

**Figure 3:**
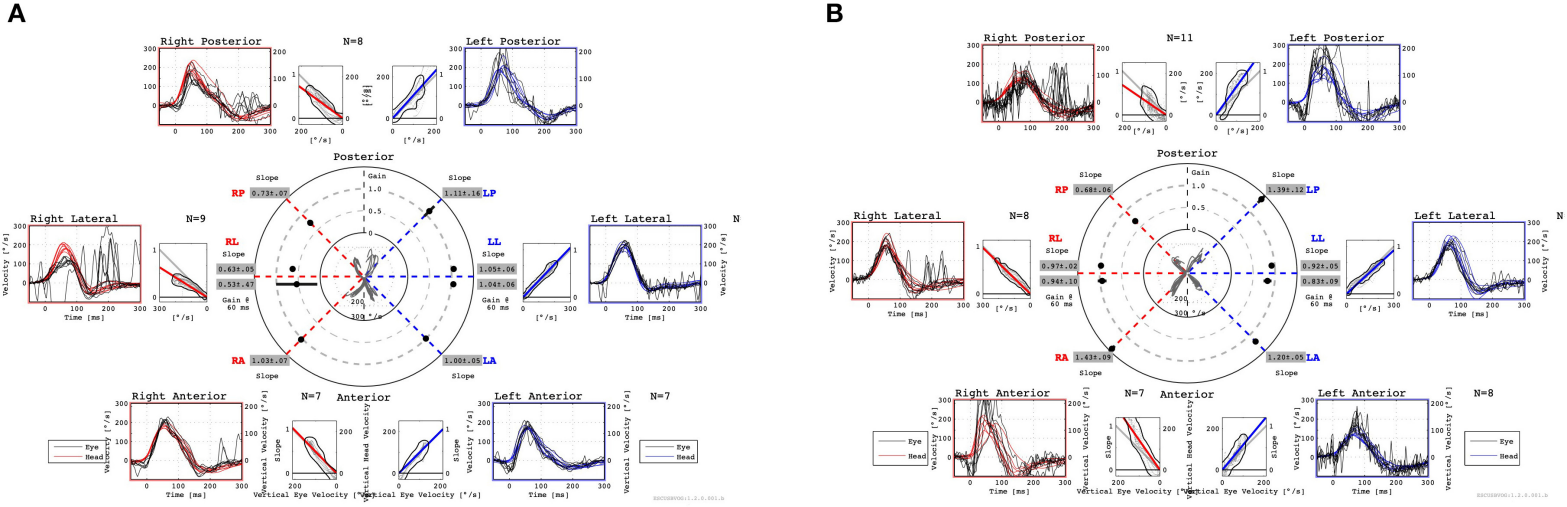
Preoperative vHIT shows a decreased VOR gain in the right LSC and PSC **(A)**. Postoperative vHIT shows that the decrease in VOR gain in the right PSC remained unchanged, but the decrease in VOR gain in the right LSC improved **(B)**. Both preoperatively and postoperatively, vHIT shows normal VOR gain in the right ASC with no change **(A, B)**. vHIT, video head impulse test; VOR, vestibulo-ocular reflex; LSC, lateral semicircular canal; PSC, posterior semicircular canal; ASC, anterior semicircular canal.

One year later, she returned to the hospital with hearing loss and severe vertigo, and her right hearing had deteriorated and scaled out (Figure 1B). The patient was again treated with prednisolone (60 mg/day tapered for 1 week), but she experienced no improvement. She was referred to our hospital to investigate the cause and received treatment for hearing loss and vertigo. At the time of the initial visit to our clinic, spontaneous nystagmus was not present. However, the head-shaking test showed constant directional nystagmus to the left. Moreover, during her vertigo attacks, as in the previous case, constant directional nystagmus to the left was observed regardless of the head position and was especially enhanced in the right lateral recumbent position. The fistula test results were negative. High-resolution computed tomography of the temporal bone revealed no pneumolabyrinths, fractures, malformations, or findings suggestive of superior canal dehiscence. Brain MRI revealed no abnormalities. Initially, we suspected MD because of the recurrent attacks of vertigo with hearing loss, tinnitus, and blocked sensation in the right ear. Contrast-enhanced MRI using HYDROPS did not show endolymphatic hydrops in the affected inner ear, which is characteristic of MD; rather, a few contrast-deficient images indicated the endolymph (Figure 2A). MD often shows a decreased response in the caloric test, which is normal in the video head impulse test (vHIT). However, in this case, the caloric test results revealed unilateral weakness in the right ear (canal paresis, CP = 50%), and the vHIT showed decreased vestibulo-ocular reflex (VOR) gain in the lateral semicircular canal (Figure 3A). She had repeated attacks of vertigo, and we considered the possibility that the PLF had progressed. Therefore, we performed an exploratory tympanotomy 4 months after her arrival at our hospital. A transcanal approach with tympanomeatal flap elevation revealed no perilymph leakage from the round window niche or surrounding footplate of the stapes. Leakages that were not visible were repaired, the mucosa was dissected, a round window was placed above and behind the footplate of the stapes, and the fissula ante fenestram was sealed with connective tissue. Middle ear lavage was collected intraoperatively andacochlin tomoprotein (CTP) detection test by monoclonal antibodies based ELISA (SRL Inc., Tokyo, Japan) revealed 30.2 ng/ml which means positive (the cutoff criteria, 30.0 ng/ml) (5). Postoperatively, the presence of PLF was confirmed. The patient's vestibular symptoms rapidly improved and nystagmus disappeared the day after surgery. Her hearing loss persisted, with no improvement. Five months postoperatively, the CP% was 52.4%, which was unchanged from the preoperative level; however, the VOR gain in the lateral semicircular canal improved in the vHIT group (Figure 3B). Six months postoperatively, contrast-enhanced MRI using HYDROPS revealed a contrast defect in the endolymph on the affected side, which was rarely observed preoperatively (Figure 2B).

## 3. Discussion

Since vertigo was induced by blowing the nose, this case was classified as Category 3 according to the Japanese PLF categorization, and the positive CTP result confirmed the diagnosis (Table 1) (1, 2). Contrast-enhanced MRI using HYDROPS showed few or no contrast deficit in the vestibule and cochlea on the affected side (Figure 2A). The mechanism of this defect is assumed to be the collapse of the endolymphatic space due to endolymph leakage at the onset of perilymph leakage and rupture of Reissner's membrane. The possibility that the rupture of Reissner's membrane in combination with PLF can cause severe hearing loss was first reported in the 1970s by Simmons as “double-membrane break syndrome” (6). Our case supports this view. The perilymphatic space is covered by a bony labyrinth that preserves the lumen even if the perilymph leaks. The perilymphatic space tends to be enlarged by the collapse of the endolymphatic space, and the contrast-enhanced perilymphatic space appears to be highlighted. After surgery, a contrast-enhanced defect on MRI revealed an endolymphatic space, which was rarely observed before surgery. The ruptured Reissner's membrane repairs itself over time, and we hypothesized that the cessation of perilymph leakage into the middle ear maintains perilymph homeostasis, resulting in gradual repair of the ruptured area (Figure 2B).

PLFs generally present with an acute onset of auditory symptoms, vestibular symptoms, or both. Sometimes, vestibular symptoms occur with fluctuating or progressive hearing loss, and as in this case, they are differentiated from MD. Therefore, in previous reports, MD and PLF were closely related, and PLF is thought to be complicated by endolymphatic hydrops, similar to MD (3, 7–9). Cerebrospinal fluid (CSF) fistula is a vestibular disorder that involves fistula as well as PLF. CSF fistula also presents with hearing loss and vestibular symptoms, similar to MD. The perilymph communicates with the CSF via the cochlear aqueduct and the CSF fistula is considered to be perilymph depletion with endolymphatic hydrop (10–13). In this case, contrast-enhanced MRI of the inner ear showed the opposite result; therefore, the fluctuating and progressive hearing loss and intermittent vestibular symptoms may be solely due to PLF.

Merchant and Schuknecht first described a histopathological entity called vestibular atelectasis, which shows the collapse of the ampulla and utricle walls (14). Eliezer et al. clinically confirmed endolymph collapse, defined as failure to visualize the utricle and at least two ampullas using delayed contrast-enhanced MRI (15). The present case had similar imaging findings and was considered to have an endolymph collapse with rupture of the Reissner's membrane. The postoperative improvement in vestibular symptoms and positive CTP suggested that there was preoperative traffic between the perilymph space and the middle ear space. In other words, preoperatively, she was thought to have a “double membrane rupture syndrome” as proposed by Simmons, in which perilymph leakage combined with rupture of Reissner's membrane causes severe hearing loss. In support of this, animal models of PLF have reported that rupture of round window alone results in mild hearing loss, but that hearing loss becomes severe when accompanied by a rupture of Reissner's membrane as well (16–19). The patient had recurrent attacks of vertigo from the age of 15 until surgery, each time the fistula was thought to have repeatedly opened and spontaneously closed. However, at age 26 and 1 year later, the patient had hearing loss along with vertigo attacks, suggesting that the Reissner's membrane had ruptured.

The patient's symptoms improved postoperatively. Even if the traffic between the perilymph space and middle ear space were surgically blocked, the rupture of Reissner's membrane would have remained. We hypothesized that the rupture of Reissner's membrane would have spontaneously repaired after surgery and that the endolymphatic collapse would have improved on imaging. Rupture and spontaneous repair of Reissner's membrane was considered the mechanism of vertigo attacks and their recovery in MD. However, this has been debated in recent years because it is not accompanied by significant hearing loss during vertigo attacks (20, 21). In fact, spontaneous repair of Reissner's membrane has not been pathologically confirmed, making this hypothesis controversial.

The patient had vestibular disorders, as determined in both the preoperative caloric test and the vHIT. Although both tests are functional tests of semicircular canals, they differ in stimulation frequency; the caloric test uses low-frequency stimulation, whereas the vHIT uses high-frequency stimulation (22, 23). Merchant and Schuknecht reported a remarkable decrease in hair cells in the vestibular atelectasis (14). Since type I hair cells are thought to be associated with high-frequency stimulation and type II hair cells with low-frequency stimulation (24), it is possible that both were disordered. Postoperatively, there was a decrease in the response to the caloric test, but the vHIT improved. This suggests that type II hair cells remained impaired, whereas type I hair cells may have been repaired. Endolymph collapse should be considered a cause of PLF symptoms.

## 4. Conclusions

The mechanisms by which PLFs cause vestibular symptoms and hearing loss remain unclear. However, in the present case, endolymph collapse on the affected side was noted on cochlear contrast-enhanced MRI. Endolymph collapse due to rupture of the Reissner's membrane may be the cause of these symptoms.

## Data availability statement

The original contributions presented in the study are included in the article/supplementary material, further inquiries can be directed to the corresponding author.

## Ethics statement

Ethical review and approval was not required for the study on human participants in accordance with the local legislation and institutional requirements. The participants provided their written informed consent to participate in this study. Written informed consent was obtained from the individual(s) for the publication of any potentially identifiable images or data included in this article.

## Author contributions

YI: Writing—original draft, Writing—review and editing. TS: Writing—review and editing. YS: Writing—review and editing. FM: Writing—review and editing. IK: Writing—review and editing.
